# Supplementary material for: tstrait: a quantitative trait simulator for ancestral recombination graphs
Source: bioRxiv. 2024 Mar 14:2024.03.13.584790. Preprint. [Version 1] doi: 10.1101/2024.03.13.584790 (PMC10980058; doi:10.1101/2024.03.13.584790)
Supplement: Supplement 1 [file NIHPP2024.03.13.584790v1-supplement-1.pdf]

bioRxiv preprint doi: https://doi.org/10.1101/2024.03.13.584790; this version posted March 14, 2024. The copyright holder for this preprint (which was not certified by peer review) is the author/funder, who has granted bioRxiv a license to display the preprint in perpetuity. It is made available under aCC-BY 4.0 International license.

Supplementary Figures

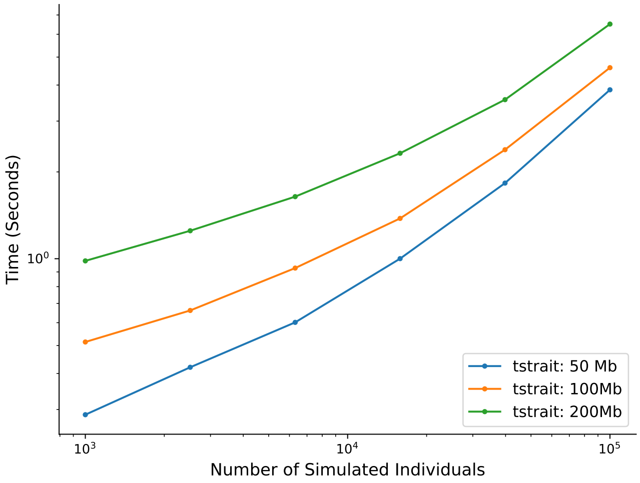

Fig. S1: Time taken to simulate quantitative traits with increasing sample size. For each sample size, we simulated an ARG under human-like parameters using the default HomSap demographic model in `stdpopsim`. Each point represents the mean time for 10 independent runs of `tstrait` for a particular ARG. The times reported are the total CPU time required to simulate a quantitative trait with 1000 causal sites, on an Intel(R) Core(TM) i9-11900H CPU and 16 GB of RAM. The trait model is a normal distribution with $\mu = 0$, $\sigma^2 = 1$, $h^2 = 0.3$, and $\alpha = 0$.
